# Systematic analysis identifies REST as an oncogenic and immunological biomarker in glioma

**DOI:** 10.1038/s41598-023-30248-0

**Published:** 2023-02-21

**Authors:** Guan Wang, Xiaxin Yang, Mei Qi, Meng Li, Meng Dong, Rui Xu, Chen Zhang

**Affiliations:** 1 Department of Pediatrics, Qilu Hospital of Shandong University, No.107 West Wenhua Road, Jinan, 250012 Shandong Province China; 2 Department of Neurology, Qilu Hospital of Shandong University, No.107 West Wenhua Road, Jinan, 250012 Shandong Province China; 3 Department of Pathology, Qilu Hospital of Shandong University, No.107 West Wenhua Road, Jinan, 250012 Shandong Province China

**Keywords:** Cancer, Cell biology, Immunology, Molecular biology, Neuroscience, Psychology, Biomarkers, Neurology, Oncology

## Abstract

The repressor element 1 silencing transcription factor (REST) has been proposed to function as a transcription factor to silence gene transcription by binding to repressor element 1 (RE1), a highly conserved DNA motif. The functions of REST in various tumors have been studied, but its role and correlation with immune cell infiltration remains uncertain in gliomas. REST expression was analyzed in datasets of The Cancer Genome Atlas (TCGA) and the Genotype-Tissue Expression (GTEx) and validated by the Gene Expression Omnibus and Human Protein Atlas databases. The clinical prognosis of REST was evaluated by clinical survival data of TCGA cohort and validated by Chinese Glioma Genome Atlas cohort. MicroRNAs (miRNAs) contributing to REST overexpression in glioma were identified by a combination of a series of in silico analyses, including expression analysis, correlation analysis, and survival analysis. The correlations between immune cell infiltration level and REST expression were analyzed by TIMER2 and GEPIA2 tools. Enrichment analysis of REST was performed using STRING and Metascape tools. The expression and function of predicted upstream miRNAs at REST and their association with glioma malignancy and migration were also confirmed in glioma cell lines. REST was highly expressed and associated with poorer overall survival and disease-specific survival in glioma and some other tumors. MiR-105-5p and miR-9-5p were identified as the most potential upstream miRNAs of REST in glioma patient cohort and experiments in vitro. REST expression was positively correlated with infiltration of immune cells and the expression of immune checkpoints such as PD1/PD-L1 and CTLA-4 in glioma. Furthermore, histone deacetylase 1 (HDAC1) was a potential REST-related gene in glioma. Enrichment analysis of REST found chromatin organization and histone modification were the most significant enriched terms, and Hedgehog-Gli pathway might be involved in the effect of REST on the pathogenesis of glioma. Our study suggests REST to be an oncogenic gene and the biomarker of poor prognosis in glioma. High REST expression might affect the tumor microenvironment of glioma. More basic experiments and large clinical trials aimed at the carcinogenetic study of REST in glioma will be needed in the future.

## Introduction

The repressor element 1 silencing transcription factor (REST), also known as NRSF or XBR, has been proposed to function as a master repressor attenuating the expression of neuronal-specific differentiation genes in neural stem cells and non-neuronal cells^1^. As a zinc-finger transcription factor, REST binds to repressor element 1 (RE1), a highly conserved DNA motif within regulatory regions to silence gene transcription. REST has been reported to target a large number of genes, including those encoding for synaptic vesicle proteins, neurotransmitter receptors, channels, transporters, and cell adhesion molecules^2^. Genome and transcriptome analyses revealed almost 2000 potential REST targets in the human genome, including both coding and noncoding genes^3^. REST was confirmed to regulate the process of neurodevelopment including neurogenesis, neuronal differentiation and maturation by repressing the expression of target genes during both in vivo and in vitro^4^. Furthermore, REST also plays a critical role in cell migration, cell replication, apoptosis, angiogenesis, and protein synthesis. Dysfunction of REST leads to disruption in gene regulatory networks, contributing to pathogenic outcomes and neurological disorders including stroke, Alzheimer’s disease, epilepsy, Parkinson’s, Huntington’s disease, Down syndrome and Prion disease^5–8^.

In the past few years, the association between REST and tumor also has aroused increasing attention. In mammary epithelial cells, REST has been proved to act as a potent tumor suppressor during several non-neural carcinomas including breast, colon, and small cell lung cancers (SCLCs)^9–11^. However, the role of REST in cell proliferation and tumor growth is not consistent depending on cell type. High expression of REST was found in aggressive neural tumors such as neuroblastoma, medulloblastoma, and glioblastoma multiforme (GBM), which was consistent with the invasiveness and severity of these tumors^12–16^. Differently, another study by gene copy number analyses showed that most gliomas expressed low amplification of REST^17^, indicating that the oncogenic role of REST still remains controversial. Thus, more studies will be needed to identify the role and underlying mechanism of REST in tumors, which may become a novel potent therapeutic target and benefit to the development of new therapeutic approaches.

Gliomas, as a heterogeneous group of primary intracranial tumors, are the most common tumors within the central nervous system (CNS) arising from glial cells. Although gliomas are sensitive to chemotherapy and radiation, these treatments are all not curative and patient survival still remains unsatisfactory, causing the need for more research to study the mechanism of gliomas as well as more creative and effective therapies. The REST seemed to be identified as a potential therapeutic target for glioma treatment. One study revealed that REST may downregulate the expression of SYN1 in glioma, thus maintaining a cancer stem-like phenotype and contributing to the development of glioma^18^. Zhang et al.^19^ found that higher REST level was significantly associated with worse clinical characteristics and poor clinical outcomes in patients suffering from glioma. A REST-associated competitive endogenous RNA (ceRNA) network including NR2F2-AS1-miR129-REST and HOTAIRM1-miR137-REST was constructed based on the TCGA and Starbase^19^. However, comprehensive research on the expression, prognosis, and mechanism of REST in glioma remains absent.

In the present study, we first performed a pan-cancer analysis of REST in multiple types of human cancer using TCGA project. The noncoding RNA (ncRNA)-associated regulation of REST expression was also explored in glioma. Then we investigated the relationship between REST expression and immune infiltration, biomarkers of immune cells, and immune checkpoints in glioma. Finally, in vitro experiments were conducted to verify the effectiveness of the predicted upstream miRNAs of REST and their regulatory relationship with REST. Taken together, our findings suggest that the protein expression level of REST correlate with clinical prognosis and tumor immune infiltration in patients with glioma.

## Methods

### Gene expression analysis

We observed the expression difference of REST between tumor and adjacent normal tissues of the TCGA project by TIMER2 (http://timer.cistrome.org/)^20^. GEPIA2 (http://gepia.cancer-pku.cn/) was also used to assess the REST expression in cancer and normal gene-expression profiling and interactive analyses based on TCGA and the Genotype-Tissue Expression (GTEx) data^21^. The UALCAN portal (http://ualcan.path.uab.edu/analysis-prot.html) was used to conduct REST protein expression analysis of the CPTAC (clinical proteomic tumor analysis consortium) dataset^22^. The GSE68848 datasets procured from the Gene Expression Omnibus database (GEO; https://www.ncbi.nlm.nih.gov/geo/) were utilized to validate the expression of REST mRNA in oligodendroglioma and astrocytoma^23^. *P* value < 0.05 was considered as statistically significant.

### Human protein atlas database

Differences in REST protein expression between glioma and normal brain tissues were examined by immunohistochemistry (IHC) images from the Human Protein Atlas (HPA) database^24^. The staining intensity evaluation was determined from the HPA (https://www.proteinatlas.org).

### Survival prognosis analysis

The “Survival Map” module of GEPIA2 was employed to conduct overall survival (OS) and disease-free survival (RFS) significance map data of REST in all TCGA tumors. Cutoff-high (50%) and cutoff-low (50%) values were used as the expression thresholds for splitting the high-expression and low-expression cohorts in OS and RFS analysis. The “Survival Analysis” module GEPIA2 was also utilized to obtain significant survival plots in glioma. The Chinese Glioma Genome Atlas (CGGA) cohort (DataSet ID: mRNAseq_693) was chosen as the validation set to conduct the OS in glioma. Samples with incomplete clinical information and overall survival < 30 days had been excluded. FPKM data from CGGA were applied for further analysis^25^. The data, such as IDH mutation status and 1p/19q codeletion, were from the study of Ceccarelli^26^. The log-rank test *P* value < 0.05 was considered as statistically significant.

### Candidate miRNA prediction and analysis

Upstream binding miRNAs of the REST were predicted by several target gene prediction programs, consisting of PITA, RNA22, miRmap, microT, miRanda, PicTar, and TargetScan. Only the predicted miRNAs that commonly appeared in more than 3 programs as mentioned above were included for subsequent analyses. These predicted miRNAs were regarded as candidate miRNAs of REST. The starBase (http://starbase.sysu.edu.cn/) was introduced to perform expression correlation analysis for miRNA-REST^27^. *P* value < 0.05 was considered as statistically significant.

### Immune infiltration analysis

TIMER2 was used to explore the infiltration level of immune cells under different copy numbers of REST and the association between REST expression and immune or cancer-associated fibroblast infiltrated in glioma. TIMER2 and GEPIA2 were both performed to analyze the correlation of REST expression with immune checkpoint expression in glioma. |R|> 0.2 and *P* value < 0.01 was considered as statistically significant.

### Enrichment analysis of REST-related partners

STRING tool (https://cn.string-db.org/) was used to obtain a total of 50 REST-binding proteins supported by experimental evidence^28^. The REST-correlated targeting genes expression in glioma was also evaluated using GEPIA2 database. The Venn web (http://bioinformatics.psb.ugent.be/webtools/Venn/) was used to conduct the intersection analysis of REST-binding genes and REST-correlated genes. Metascape tool (https://metascape.org/gp/index.html#/main/)was used to conduct the pathway and gene ontology (GO) analysis of REST-related partners^29^.

### Cell lines and culture

Human cell lines U251 and T98G were purchased from ATCC and cultured in Dulbecco’s modified Eagle’s medium (DMEM) (Invitrogen Thermo Fisher Scientific, USA). Culture media contained 10% (vol/vol) fetal bovine serum (FBS), 100 units/ml penicillin and 0.1 mg/ml streptomycin. The above cells were all incubated in an incubator at 37 °C with 5% CO_2_.

### Cell transfection

The mimics of miRNAs were obtained from GenePharma Co., Ltd (Shanghai, China). The primers of all genes were synthesized by TsingKe Biological Technology (Beijing, China). Transfection of miRNAs was performed using Lipofectamine 3000 (Invitrogen, Carlsbad, CA, USA) when the cell density reached approximately 50–70% (usually 24 h after inoculation) following the instruction of manufacture. The sequences of the mimics were as follows: miR-105-5p mimics (5′-UCAAAUGCUCAGACUCCUGUGGU-3′, and 5′-CACAGGAGUCUGAGCAUUUGAUU-3′), miR-9-5p mimics (5′-UCUUUGGUUAUCUAGCUGUAUGA-3′ and 5′-AUACAGCUAGAUAACCAAAGAUU-3′) and negative control (NC, 5′-UUCUCCGAACGUGUCACGUTT-3′and 5′-ACGUGACACGUUCGGAGAATT-3′).

### Real-time quantitative PCR

Briefly, total RNA was extracted from cells using the RNA fast extraction kit (Cat# 220010, Fastagen, Shanghai, China). Real-time amplification was achieved using the ABI 7900HT Fast real-time PCR system (Applied Biosystems, Foster City, CA) and quantified by SYBR® Green-based gene expression analysis. The primers employed for real-time quantitative PCR were as follows: miR-105-5p (5′-AACCTCGCTCAAATGCTCAGA-3′, and 5′-TATGCTTGTTCACGACACCTTCAC-3′), miR-9-5p (5′-AGCCTCTCTCCTCTTTGGTTATCT-3′ and 5′-TATGGTTGTTCTGCTCTCTGTGTC-3′), REST (5′-GCCGCACCTCAGCTTATTATG-3′ and 5′-CCGGCATCAGTTCTGCCAT-3′), β-actin (5′-GACAGGATGCAGAAGGAGATTACT-3′ and 5′-TGATCCACATCTGCTGGAAGGT-3′).

### Western blot

Total protein was quantified using the bicinchoninic acid (BCA) method and separated using 8% SDS-PAGE. Then the gels were transferred to PVDF membranes. After blocked with 5% skim milk in TBST, the membranes were incubated with the primary antibodies: REST (Proteintech, China) and β-actin (Sigma, USA) overnight at 4 °C. After washing with TBST, the membranes were reacted with the secondary antibody. Finally, detection and quantification were achieved with the Li-Cor Odyssey imaging system and ImageJ software.

### CCK8 assay

The proliferative capacity of glioma cells was determined using the CCK8 kit (DOJINDO, Japan). After cell transfection, log phase grown cells were implanted in 96-well-plates with 5000 cells per well. At 0, 24, 48, and 72 h post-transfection, 10 µl CCK-8 reaction solution was added to each well. The plates were then placed back in the incubator for 1 h. Finally, the absorbance of each well was measured at 450 nm using a multifunctional enzyme marker.

### Wound healing assay

After cell transfection, cells were digested and resuspended with serum-free DMEM culture medium, inoculated into 6-well plates at a cell count of 150,000 per well, and cell transfection was performed at 24 h. When the cells were grown to fusion (usually 48 h after inoculation), a 200 µl pipette tip was used to make a vertical “wound” in the cell layer. This was followed by incubation with DMEM containing 0.5% FBS to eliminate the effect of proliferation on the migratory phenotype. Image J software was applied to calculate the scratch healing area. Scratch healing ratio of X h = (0 h scratch area–X h scratch area)/0 h scratch area.

### Immunohistochemistry (IHC) staining

We further validated the protein expression level of REST and CD4 in an independent group of 10 patients with glioma receiving operation at Qilu Hospital of Shandong University by IHC. Brain tissues from 3 normal subjects were used as controls. The validation was approved by the Ethics Committee of Qilu Hospital, and all patients provided informed consent. IHC staining was performed as previously described^30^. Serial sections of formalin-fixed tissues were deparaffinized, rehydrated, immersed in citrate buffer (pH 6.0), and microwaved for 15 min to retrieve antigens. Slides were stained for 1 h with rabbit anti-REST (Proteintech, China) and mouse anti-CD4 (Proteintech, China). Images were captured with an Olympus microscope. The histochemical scoring (H-SCORE) was employed to measure the expression level of target proteins. Each slide stained for REST or CD4 was individually reviewed and scored by two independent observers, who were blinded to the clinicopathologic data. Discrepancies in scoring between the two observers were resolved by additional review of the specimens and discussion between the reviewers until consensus was achieved.

### Statistical analysis

For the data with normal distribution, we used the unpaired Student’s t test for comparison between two groups and one-way ANOVA followed by Bonferroni test for multiple group comparison. For non-normally distributed data, Wilcoxon test was utilized to make the comparison between two groups. Spearman correlation test was used to analyze the correlation of gene expression. The Log-rank test was employed to generate and compare Kaplan–Meier curves. *P* < 0.05 was considered statistically significant.

## Results

### Pan-cancer analysis of REST expression

**Figure 1:**
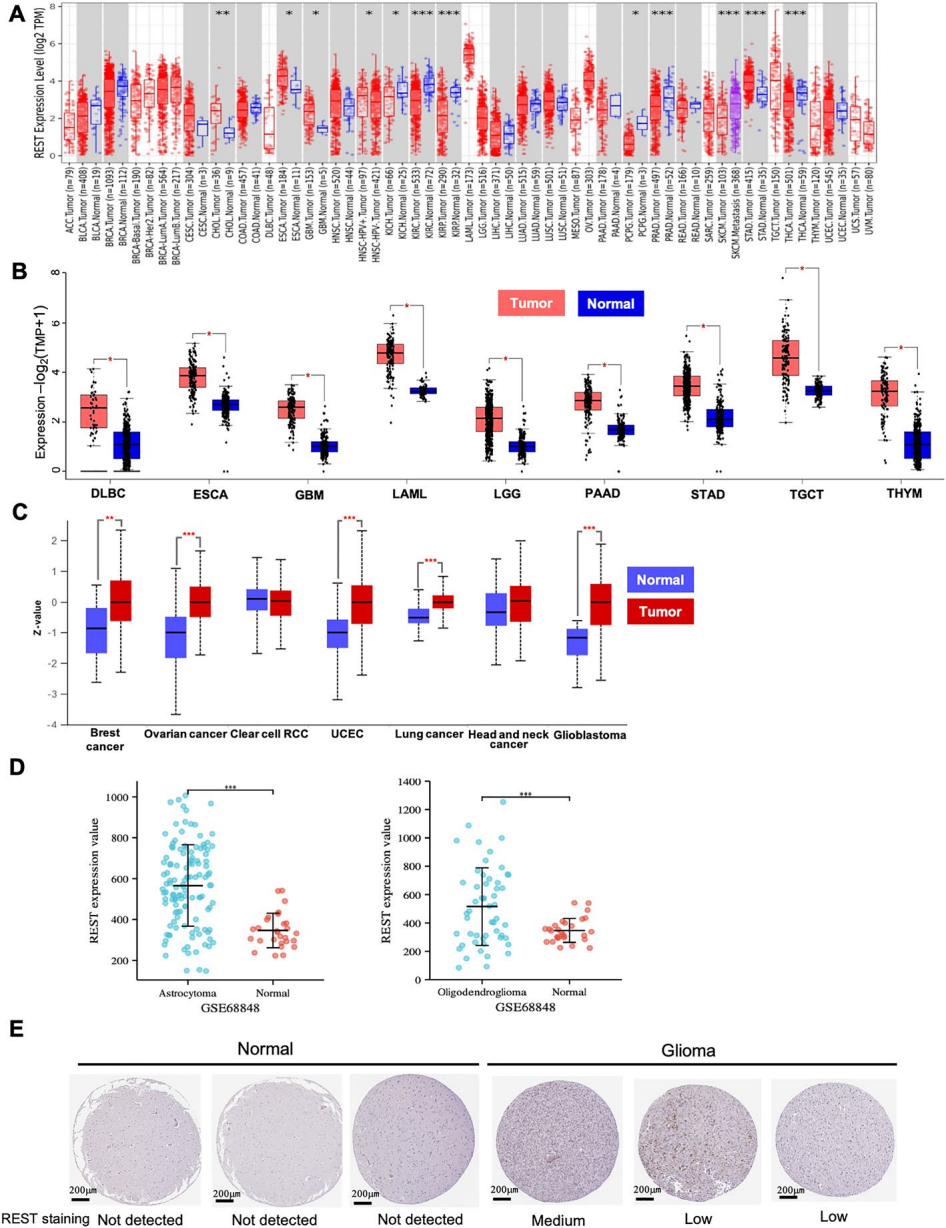
Pan-cancer analysis of REST expression. (**A**) The expression of REST mRNA in human cancers and normal tissues of the TCGA cohort by TIMER2 (http://timer.cistrome.org/). (**B**) REST mRNA expression in cancer tissues compared with corresponding TCGA and GTEx normal tissues by GEPIA2 (http://gepia.cancer-pku.cn/). (**C**) The expression of REST total protein in breast cancer, ovarian cancer, GBM, UCEC and LUAD by UALCAN portal (http://ualcan.path.uab.edu/analysis-prot.html). (**D**) REST mRNA expression in oligodendroglioma and astrocytoma of GSE68848 datasets by GraphPad Prism 9.3.1 (https://www.graphpad.com/). (**E**) The representative immunohistochemical images of REST in glioma and normal tissues by HPA (https://www.proteinatlas.org). *P value < 0.05; **P value < 0.01; ***P value < 0.001.

We first assessed the REST expression in pan-cancer data of TCGA. The analysis results revealed the higher REST mRNA expression in five tumors, including CHOL, ESCA, GBM, HNSC-HPV^+^ (compared to HNSC-HPV^−^) and STAD, while lower expression was observed in KICH, KIRC, THCA, KIRP, PCPG and PRAD (Fig. 1A, tumor abbreviations in Table S1). After including the normal tissue of the GTEx dataset as controls, the expression of REST mRNA was significantly upregulated in 9 cancer types, including DLBC (tumor 47 and normal 337), ESCA (tumor 182 and normal 286), GBM (tumor 163 and normal 207), LAML (tumor 173 and normal 70), LGG (tumor 518 and normal 207), PAAD (tumor 179 and normal 171), STAD (tumor 408 and normal 211), TGCT (tumor 137 and normal 165) and THYM (tumor 118 and normal 339) (Fig. 1B, *P* < 0.05). The higher expression of REST total protein in the primary tissues of breast cancer (tumor 125 and normal 18, *P* < 0.01), ovarian cancer (tumor 100 and normal 25, *P* < 0.001), GBM (tumor 99 and normal 10, *P* < 0.001), UCEC (tumor 100 and normal 31, *P* < 0.001) and LUAD (tumor 111 and normal 111, *P* < 0.001) than in normal tissues was shown by the results of the CPTAC dataset (Fig. 1C).

### The REST expression in glioma

To further verify the expression level of REST in glioma, we tested REST mRNA expression in the GEO dataset between oligodendroglioma (glioma 53 and normal 28) and astrocytoma (glioma 123 and normal 28). The analysis results revealed the higher REST mRNA expression in oligodendroglioma and astrocytoma compared with normal tissues (Fig. 1D, *P* < 0.001). We also confirmed the expression level of REST protein by immunohistochemical analysis in HPA database. Similarly, the representative results showed that higher expression of REST was observed in glioma samples, whereas REST was not detected in normal samples (Figs. 1E and S1). These results confirmed that REST was highly expressed in glioma.

### The prognostic values of REST in human cancer

**Figure 2:**
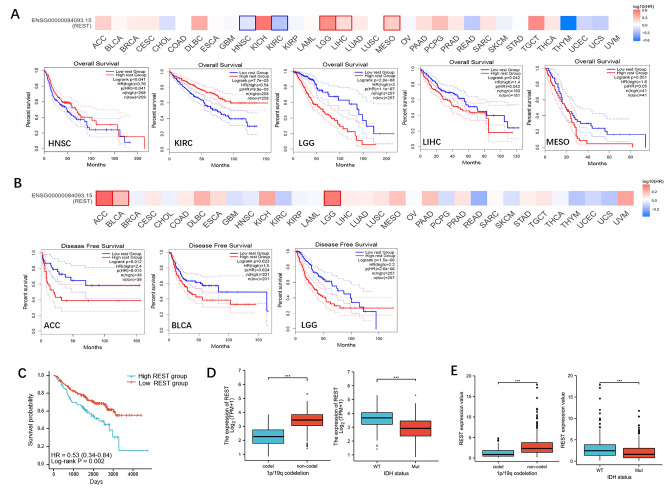
The overall survival (OS) and disease-free survival (RFS) analysis for REST in human cancers. (**A**) The overall survival map and Kaplan–Meier curves with positive results of different tumors in TCGA cohort with different REST gene expression by GEPIA2 (http://gepia.cancer-pku.cn/). (**B**) The disease-free survival map and Kaplan–Meier curves with positive results of different tumors in TCGA cohort with different REST gene expression by GEPIA2 (http://gepia.cancer-pku.cn/). (**C**) The Kaplan–Meier curves of glioma in CGGA cohort with different REST gene expression by GraphPad Prism 9.3.1 (https://www.graphpad.com/). log-rank test *P* value < 0.05. (**D**) The REST mRNA expression level in non-1p/19q codeletion or IDH status-WT gliomas in TCGA cohort by GraphPad Prism 9.3.1 (https://www.graphpad.com/). (**E**)The REST mRNA expression level in non-1p/19q codeletion or IDH status-WT gliomas in CGGA cohort by GraphPad Prism 9.3.1 (https://www.graphpad.com/). ***P value < 0.001.

Two prognostic indices, OS and RFS, were included to conduct the survival analysis of REST in cancers. For OS, high expression of REST in LGG and LIHC had an unfavorable prognosis, while KIRC and HNSC patients with higher expression of REST indicated a better prognosis (Fig. 2A, *P* <  0.05). For RFS, increased expression of REST indicated poor prognosis in ACC, BLCA, and in LGG (Fig. 2B, *P* <  0.05). Furthermore, we confirmed the prognostic value of REST in CGGA cohort. The results showed that higher expression of REST in glioma had a poorer prognosis of overall survival (Fig. 2C, *P* = 0.002), which was consistent with Fig. 2A,B. Otherwise, the REST mRNA expression level was higher in non-1p/19q codeletion or IDH status-WT gliomas in both TCGA cohort (Fig. 2D, *P* <  0.001) and CGGA cohort (Fig. 2E, *P* <  0.001). These results suggested the oncogenic role of REST in glioma.

### Prediction and analysis of upstream miRNAs of REST

**Figure 3:**
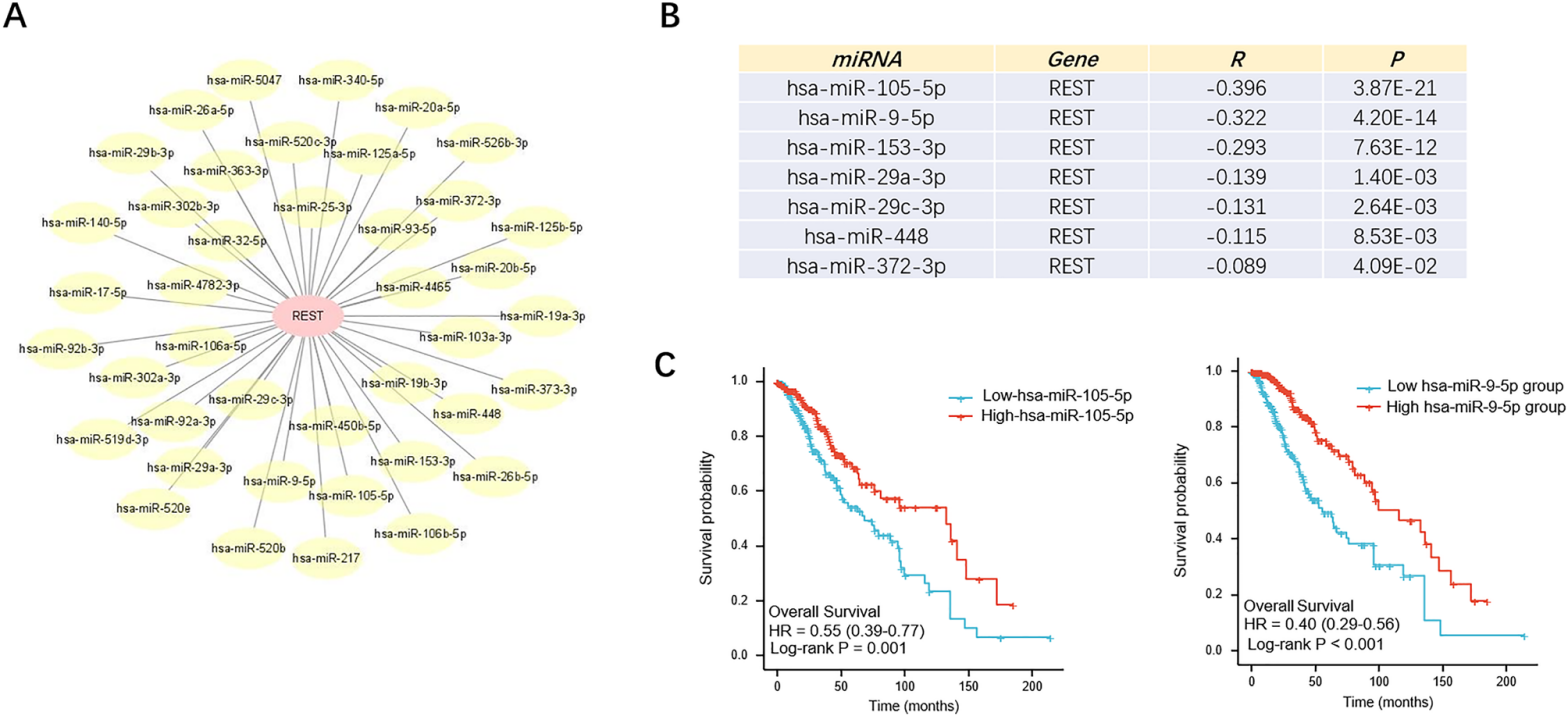
Identification of potential upstream miRNAs of REST in glioma. (**A**) The miRNA-REST regulatory network established by cytoscape 3.8.0 software (https://cytoscape.org/). (**B**) The expression correlation of predicted miRNAs and REST in glioma assessed by StarBase (http://starbase.sysu.edu.cn/). (**C**) The Kaplan–Meier curves of predicted miRNAs in glioma in TCGA cohort by GraphPad Prism 9.3.1 (https://www.graphpad.com/). log-rank test *P* value < 0.05.

It has been widely acknowledged that ncRNAs are responsible for the regulation of gene expression. To ascertain whether REST was modulated by some ncRNAs, we first predicted upstream miRNAs that could potentially bind to REST and finally found 41 miRNAs. To improve visualization, a miRNA-REST regulatory network was established using cytoscape software (Fig. 3A). Based on the action mechanism of miRNA in the regulation of target gene expression, there should be a negative correlation between miRNA and REST. Thus, the expression correlation analysis showed that 7 of 41 miRNAs were significantly negatively correlated with the REST in glioma (Fig. 3B, *P* <  0.05). Additionally, the prognostic values of the 7 miRNAs in glioma were determined. As shown in Fig. 3C, miR-105-5p and miR-9-5p were markedly downregulated in glioma and their upregulations were positively linked with patients’ prognosis (*P* < 0.05). And the other 5 miRNAs showed no significant correlation with patients’ prognosis in glioma (Fig. S2). All these findings suggest that miR-105-5p and miR-9-5p might be the potential regulatory miRNAs of REST in glioma.

### REST positively correlates with immune cell infiltration in glioma

**Figure 4:**
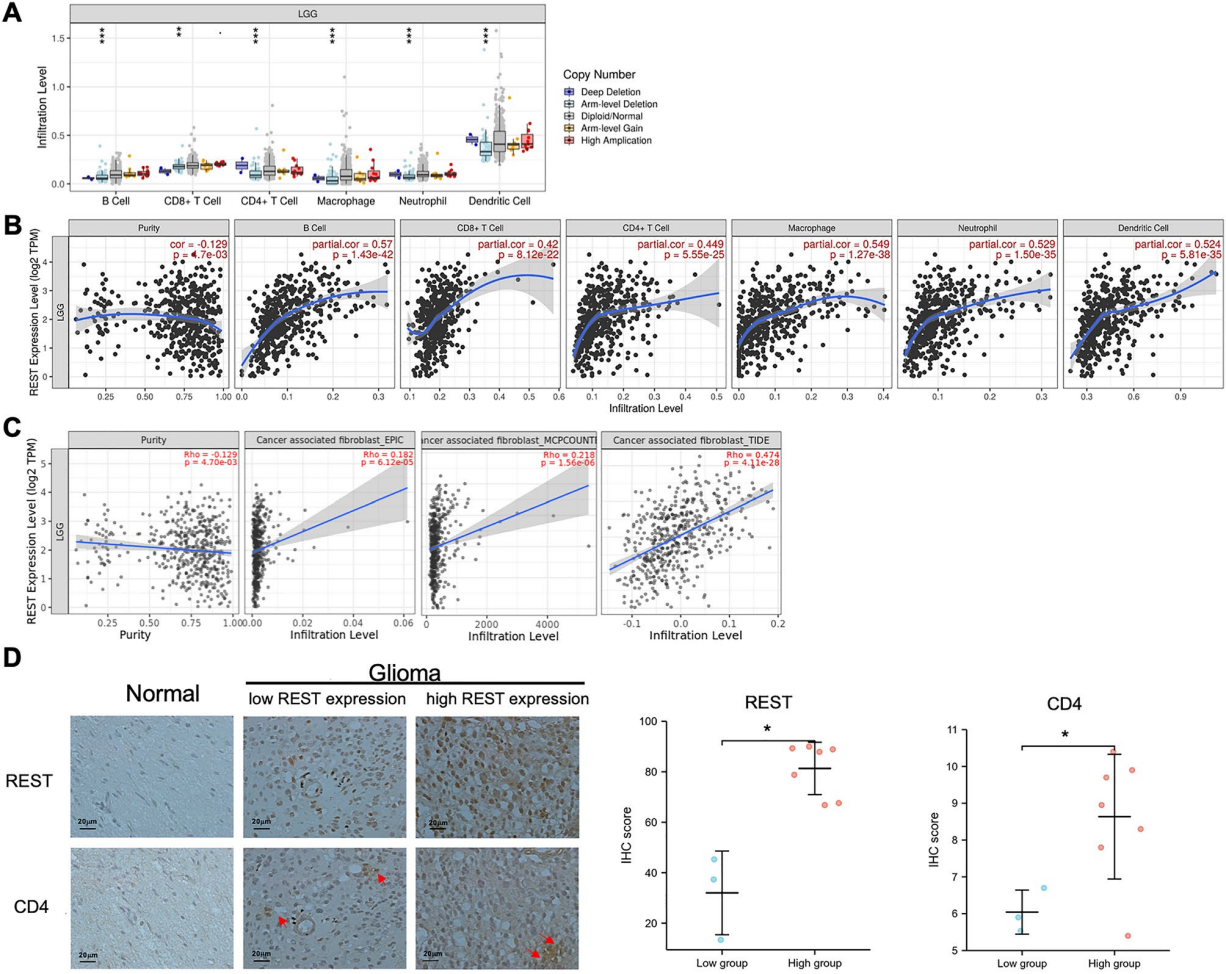
The correlation between REST and immune cell infiltration in glioma. (**A**) The infiltration level of various immune cells under different copy numbers of REST in glioma by TIMER2 (http://timer.cistrome.org/). (**B**) The correlation between REST expression level and infiltration level of immune cells in glioma by TIMER2 (http://timer.cistrome.org/). (**C**) The correlation between REST expression level and infiltration level of cancer-associated fibroblasts in glioma based on the EPIC, MCPCOUNTER and TIDE algorithms by TIMER2 (http://timer.cistrome.org/). (**D**) IHC staining (Olympus microscope) and the distribution of REST and CD4 IHC scores (H-SCORE, GraphPad Prism 9.3.1) in normal and glioma tissues by different REST expression. All data are shown as mean ± SD, *P value < 0.05; **P value < 0.01; ***P value < 0.001.

Tumor-infiltrating immune cells, as prominent components of the tumor microenvironment, were closely linked to the initiation, progression, or metastasis of cancer. As shown in Fig. 4A, a significant change of immune cell infiltration level in chromosome arm-level deletion mutation at REST compared with the normal was observed in glioma using a two-sided Wilcoxon rank-sum test. Then, the correlation of REST expression level with immune cell infiltration level was evaluated. As presented in Fig. 4B, REST expression was significantly associated with all analyzed immune cells, including B cells, CD8^+^ T cells, CD4^+^ immune cells, macrophages, neutrophils, and dendritic cells in glioma (*P* < 0.001). Cancer-associated fibroblasts in the stroma of the tumor microenvironment were reported to participate in modulating the function of various tumor-infiltrating immune cells. As shown in Fig. 4C, the REST expression level in glioma is also positively correlated with the infiltration level of cancer-associated fibroblasts based on the EPIC, MCPCOUNTER and TIDE algorithms (*P* < 0.001). Moreover, IHC staining was conducted to evaluate the expression of REST and CD4 in normal and glioma tissues with different REST expression. IHC showed that the expression of CD4 was up-regulated in the high-REST subgroup (Fig. 4D, *P value < 0.05).

### Expression correlation of REST and biomarkers of immune cells in glioma

To further explore the role of REST in tumor immune, we determined the expression correlation of REST with biomarkers of immune cells in glioma. As listed in Table 1, REST was significantly positively correlated with B cell’s biomarkers (CD19 and CD79A), CD4^+^ immune cell’s biomarker (CD4), M1 macrophage’s biomarkers (IRF5 and PTGS2), M2 macrophage’s biomarkers (CD163, VSIG4, and MS4A4A), neutrophil’s biomarkers (ITGAM), and dendritic cell’ s biomarkers (HLA-DPB1, HLA-DQB1, HLA-DRA, HLA-DPA1, CD1C, NRP1, and ITGAX) in glioma. These findings partially support that REST is positively linked to immune cell infiltration. |R|> 0.2 and *P* value < 0.01 was considered as statistically significant.

**Table 1:** Correlation analysis between REST and biomarkers of immune cells in glioma determined by GEPIA database.

Immune cell	Biomarker	R value	*P* value
B cell	CD19	0.37^a^	7.90E−19^a^
	CD79A	0.25^a^	1.10E−08^a^
CD8^+^T cell	CD8A	0.16	2.70E−04^a^
	CD8B	0.087	4.80E−02^a^
CD4^+^T cell	CD4	0.59^a^	8.60E−51^a^
M1 microphage	NOS2	0.0016	9.70E−01
	IRF5	0.55^a^	2.00E−42^a^
	PTGS2	0.22^a^	3.50E−07^a^
M2 microphage	CD163	0.36^a^	5.10E−17^a^
	VSIG4	0.56^a^	6.20E−44^a^
	MS4A4A	0.49^a^	2.60E−32^a^
Neutrophil	CEACAM8	0.098	2.60E−02
	ITGAM	0.64^a^	2.60E−60^a^
	CCR7	0.2	3.10E−06
Dendritic cell	HLA-DPB1	0.48^a^	2.80E−31^a^
	HLA-DQB1	0.3^a^	5.10E−12^a^
	HLA-DRA	0.55^a^	8.10E−43^a^
	HLA-DPA1	0.5^a^	2.30E−34^a^
	CD1C	0.23^a^	7.80E−08^a^
	NRP1	0.23^a^	7.40E−08^a^
	ITGAX	0.5^a^	1.20E−33^a^

^a^These results are statistically significant.

### Relationship between REST and immune checkpoints in glioma

**Figure 5:**
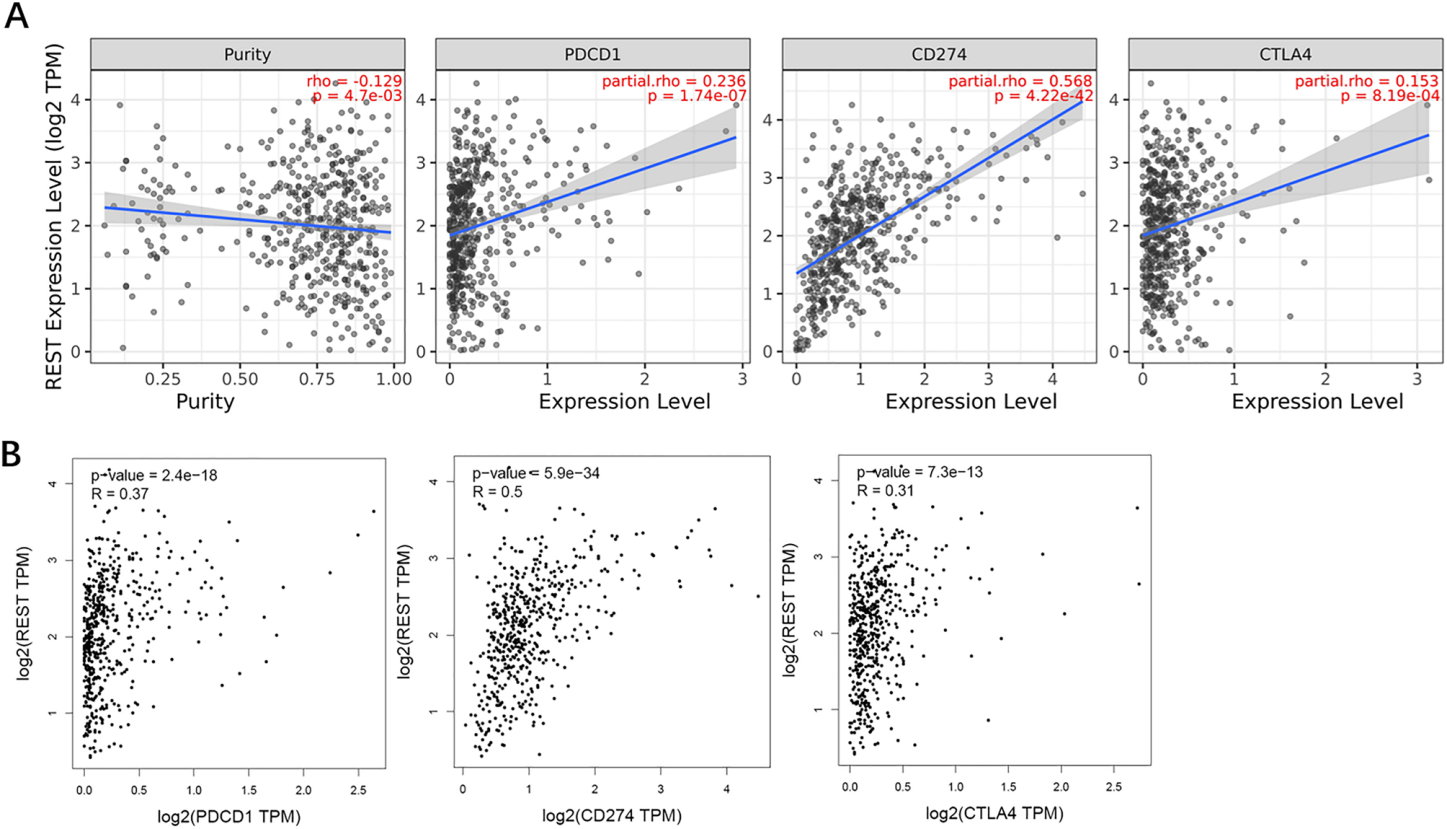
Relationship between REST and immune checkpoints in glioma. (**A**) Spearman correlation of REST with expression of PD-1, PD-L1, and CTLA-4 in glioma by TIMER2 (http://timer.cistrome.org/). (**B**) The expression correlation of REST with PD-1, PD-L1, and CTLA-4 in glioma by GEPIA2 (http://gepia.cancer-pku.cn/).

PD1/PD-L1 and CTLA-4 are important immune checkpoints that are responsible for tumor immune escape. Considering the potential oncogenic role of REST in glioma, the relationship of REST with PD1, PD-L1, or CTLA-4 was assessed. We found that the expression level of REST protein was significantly positively correlated with PD1, PD-L1, and CTLA-4 in glioma, which was adjusted by purity (Fig. 5A, *P* <  0.001). Similar to TIMER data analysis, there was significant positive correlation of REST with PD1, PD-L1, or CTLA-4 in glioma by GEPIA2 (Fig. 5B, *P* < 0.001). These results demonstrate that tumor immune escape might be involved in REST mediated carcinogenesis of glioma.

### Enrichment analysis of REST-related partners

**Figure 6:**
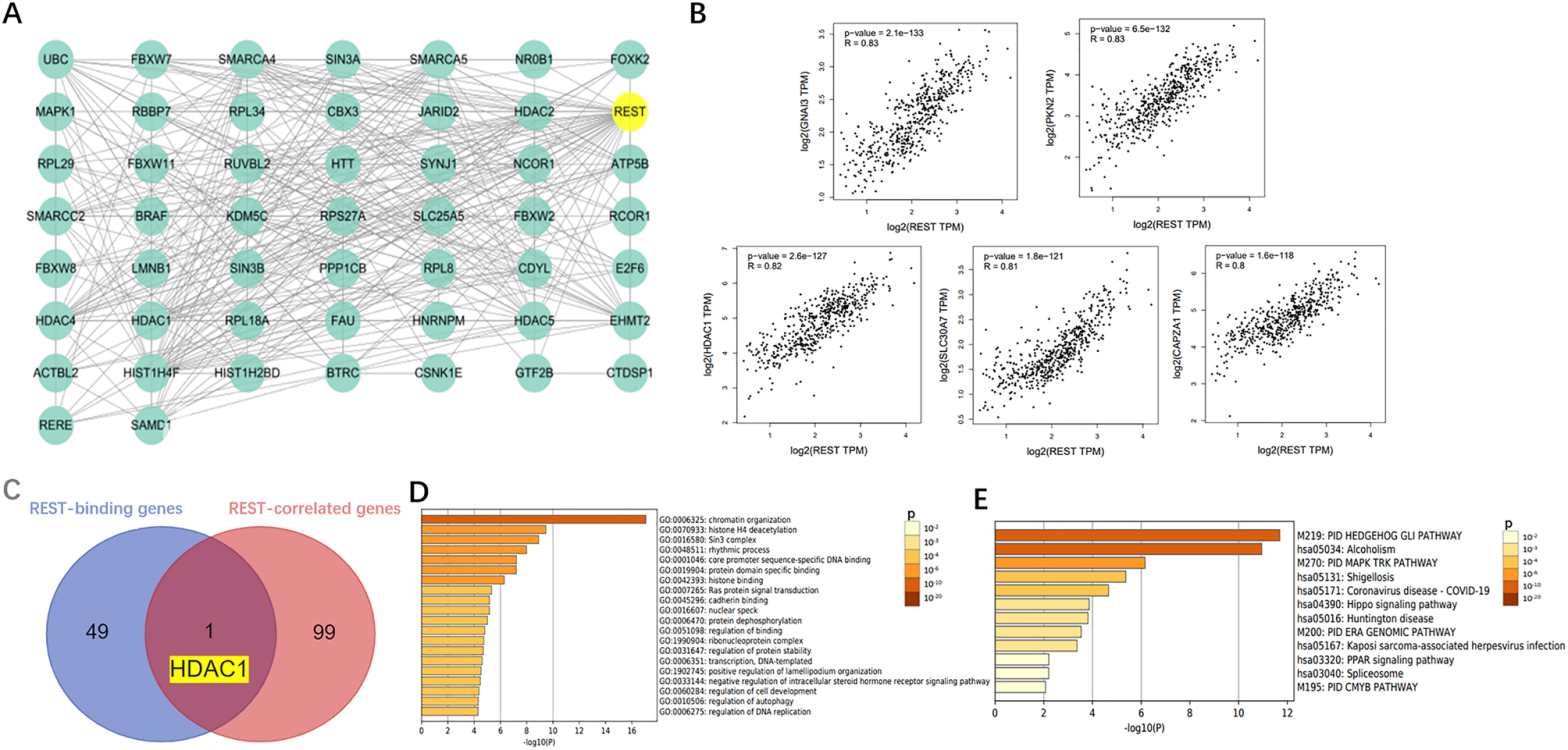
Enrichment analysis of REST-related partners. (**A**) The REST-binding proteins assessed by the STRING tool (https://cn.string-db.org/). (**B**) The expression correlation between REST and top 5 REST-correlated genes in glioma by GEPIA2 (http://gepia.cancer-pku.cn/). (**C**) An intersection analysis of the REST-binding genes and REST-correlated genes by Venn web (http://bioinformatics.psb.ugent.be/ webtools/Venn/). (**D**,**E**) GO analysis or KEGG and canonical pathways of the REST-related genes by Metascape tool (https://metascape.org/gp/index.html#/main/).

To further investigate the molecular mechanism of the REST gene in tumorigenesis, we attempted to screen out the targeting REST-binding proteins and the REST expression-correlated genes by a series of pathway enrichment analyses. We used the TRING tool to obtain a total of 50 REST-binding proteins, which were supported by experimental evidence (Fig. 6A). And then we used the GEPIA2 tool to obtain the top 100 genes that correlated with REST expression in glioma and the top 5 genes were shown in Fig. 6B (*P* < 0.001). An intersection analysis of the above two groups showed one common member, HDAC1 (Fig. 6C). Gene ontology enrichment and pathway analyses of REST-related partners were also provided. Accumulative hypergeometric p-values and enrichment factors were calculated and used for filtering. The GO analysis results were combined with molecular functions, biological processes, and cellular components. As shown in Fig. 6D,E, chromatin organization and histone modification were the most significant enriched terms, and Hedgehog-Gli pathway might be involved in the effect of REST on the pathogenesis of glioma (*P* < 0.01).

### Experimental validation

**Figure 7:**
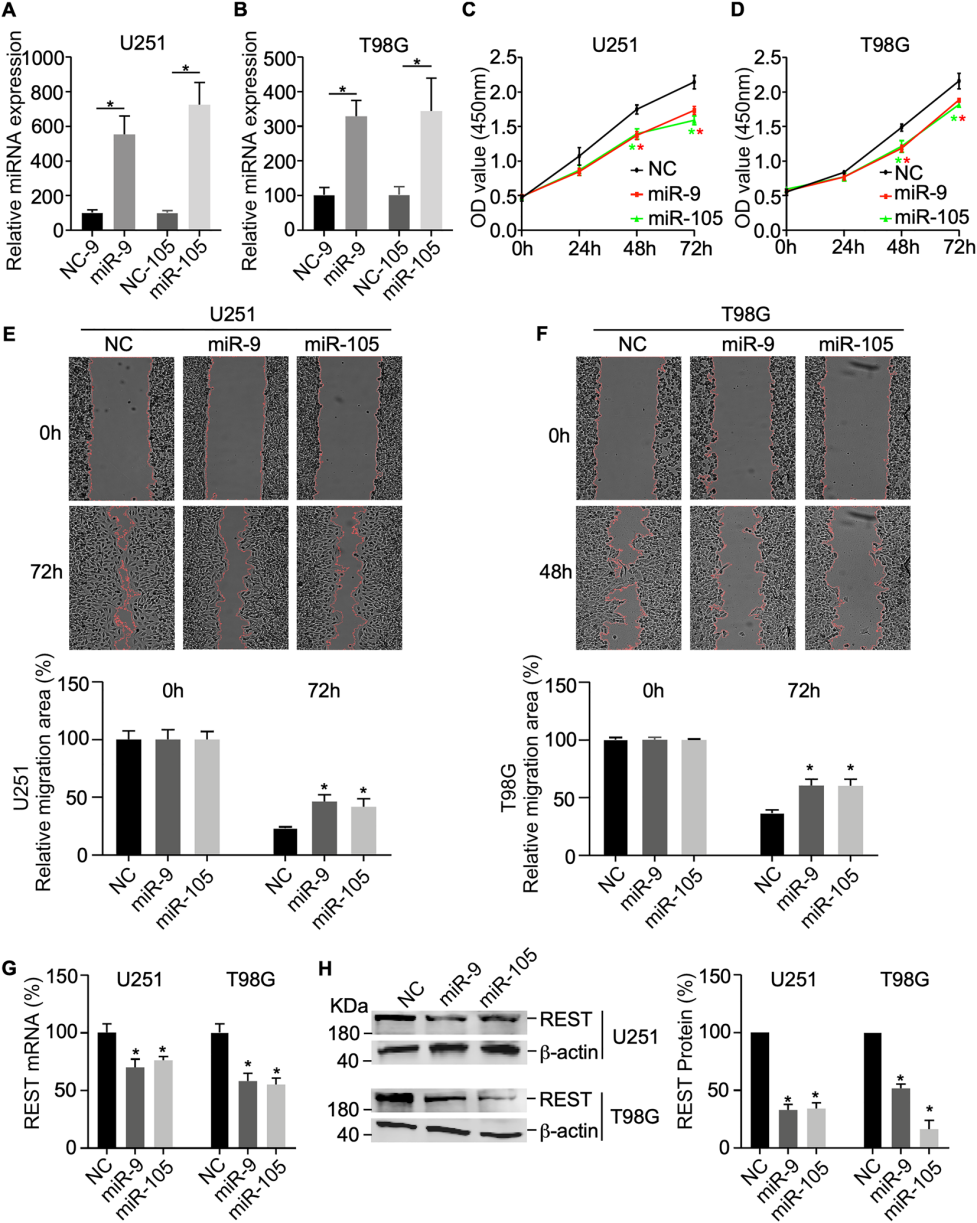
Experimental validation between REST and miRNAs. (**A**,**B**) The miRNA expression of miR-105-5p and miR-9-5p by transfection with miRNA-mimics in U251 and T98G cell lines. (**C**,**D**) Cell growth and proliferation were determined by CCK8 assay (**E**,**F**) Cell migration was determined by wound healing assay. The expression levels of REST mRNA (**G**) and protein (**H**) were determined by qRT-PCR and Western Blot (GraphPad Prism 9.3.1, https://www.graphpad.com/ and ImageJ V1.8.0 software, https://imagej.nih.gov/ij/index.html). These results are representative of three independent experiments with similar results. All data are shown as mean ± SD, * *P* value < 0.05.

As it is difficult to get hold of glioma cells, we performed in vitro experiments with REST in U251 and T98G cell lines to verify the effectiveness of the predicted upstream miRNAs of REST and their regulatory relationship. MiR-105-5p and miR-9-5p were overexpressed by miRNA-mimics and their own expression efficiency was detected. The results showed that the levels of miRNAs expression were significantly increased in U251 and T98G cell lines (Fig. 7A,B, *P* < 0.05). CCK8 assay was used to explore the role of miRNAs in the proliferation of glioma cells. Figure 7C,D showed that miR-105-5p and miR-9-5p can significantly reduce the proliferation in glioma cells. We performed a wound healing assay to investigate the migration ability of miRNAs, and the results showed that the two miRNAs significantly inhibited the migration of U251 and T98G cell lines (Fig. 7E,F). Moreover, the silencing efficiency of REST mRNA and protein regulated by miRNAs were evaluated by qRT-PCR (Fig. 7G) and Western Blot (Fig. 7H). The results indicated that miR-105-5p and miR-9-5p can effectively regulate the expression levels of REST mRNA and protein (*P* < 0.05).

## Discussion

Emerging studies have shown that REST play key roles in the initiation and development of multiple human cancers^9,11,31^. In this study, we found an upregulation of REST expression in 9 cancer types and the prognostic value of REST in 6 cancer types, suggesting an oncogenic function of REST in tumors, especially in gliomas. We observed that REST expression was positively correlated with immune infiltration levels and the expression of immune checkpoint markers in glioma. After performing correlation analysis, expression analysis, survival analysis, and experiments in vitro, we identified miR-105-5p and miR-9-5p as the potential upstream miRNAs of REST in glioma. Our results strongly suggested that REST may be used as a potential prognostic and immunological biomarker in glioma.

In this study, we found REST was highly expressed in most tumors, such as DLBC, ESCA, GBM, LAML, LGG, PAAD, STAD, TGCT, and THYM compared to normal tissues. Survival analysis of REST indicated the consistence between high expression of REST with poor OS and RFS LGG patients. Kaplan–Meier analyses in CGGA cohort also suggested an association between high REST expression and poor OS in glioma. Furthermore, high REST expression was positively correlated with IDH status-WT and non-1p/19q codeletion. Therefore, it is possible that REST may play an oncogenic role in glioma. However, these findings challenge some research on neural carcinoma, which indicated a downregulated or normal expression of REST in tumor tissues^17,18,32^. Therefore, the knowledge of REST in tumors, especially in glioma, remains inadequate and needs to be further investigated.

A growing body of evidence indicates that ncRNAs, including miRNAs, circular RNAs (circRNAs), and long non-coding RNAs (lncRNAs), regulate the expression of gene by interacting with each other through the ceRNA mechanism, thus influencing the progression of cancer^33,34^. In the current study, miR-9-5p and miR-105-5p were identified as the potential upstream regulatory miRNAs of REST in glioma and their upregulation were positively linked to patients’ prognosis. Furthermore, the results in vitro revealed the significant downregulation of REST mRNA and protein levels and the inhibition of proliferation and migration in U251 and T98G cell lines by over-expressed miR-9-5p and miR-105-5p. These results confirmed miR-9-5p and miR-105-5p as the most potential tumor suppressive miRNAs of REST in adult type diffuse glioma, which included IDH-mutant, 1p/19q codeleted oligodendroglioma; IDH-mutant, non-codeleted astrocytoma; and IDH-wildtype glioblastoma^35^. Reports showed the inhibitory roles of miR-105-5p in the proliferation and migration of glioma cells, while the regulatory mechanism during the process is still undefined^36,37^. Previous studies have found that miR-9-5p could interact with REST and regulate its gene expression^38,39^. Taken together, miR-9-5p and miR-105-5p/REST axis were identified as potential regulatory pathways in glioma, representing a novel potential treatment strategy for glioma.

Numerous studies have indicated that immune cell infiltration could influence the prognosis and immunotherapy of cancer patients^40^. However, there are only few studies focus on the association of REST with immune infiltration in tumors. Our results first suggested that REST expression was significantly positively correlated with immune cells, including B cells, CD8^+^ T cells, CD4^+^ immune cells, macrophages, neutrophils, and dendritic cells in glioma, as well as the particular biomarkers of these immune cells. The infiltrative immune cells suppress normal immune function in the tumor microenvironment (TME), preventing tumor cells from immune-mediated killing and thus accelerating tumor growth^41^. Overall, these findings indicated that REST may alter tumor immune microenvironment, and immune cell infiltration might participate in REST-mediated oncogenic roles in glioma.

Furthermore, the efficacy of immunotherapy also depends on the activation of immune checkpoints, such as programmed cell death-1 (PD-1), programmed cell death-ligand 1 (PD-L1), and cytotoxic T lymphocyte-associated antigen-4 (CTLA-4). Previous studies have revealed the important role of PD-1/PD-L1 axis in progression and immunotherapies of glioma^42^. Liu et al. found that the level of serum soluble CTLA-4 (sCTLA-4) was associated with the pathogenesis and progression of glioma, which might become a valuable predictor of the development and prognosis in glioma^43^. Our founding showed that high expression of REST was positively linked to PD1, PD-L1, and CTLA-4 in glioma. Taken together, these results suggest that REST may mediate the carcinogenesis of glioma by the mechanism of tumor immune escape, and targeting REST might increase the efficacy of immunotherapy in glioma.

In this study, we presented evidence of histone deacetylase 1 (HDAC1) as a potential REST-binding protein and the REST expression-correlated gene in glioma through a series of pathway enrichment analyses. HDAC1 primarily localizes in the nucleus, regulating the gene expression level through deacetylation of histones or transcription factors^44^. HDAC1/REST had been identified a transcriptional complex to repress gene transcription^45,46^. Fan et al. found the critical role of HDAC1 in glioma, as both a prognostic and immune infiltration biomarker and a key component of the HDAC1-related signature for accurate prognosis prediction^47^. Based on the existing research and our findings, we infer that HDAC1/REST interaction may participate in glioma and the mechanisms of miR-9-5p and miR-105-5p/HDAC1/REST axis in glioma are needed to be validated.

Furthermore, our enrichment and pathway analyses indicated that REST potentially impacts the etiology or pathogenesis of glioma might by functioning in chromatin organization, histone H4 deacetylation, and Sin3 complex. These results are consistent with previous articles, indicating that REST mediated chromatin remodeling^48^, caused cell death by inducing histone H4 deacetylation^49^ and recruited Sin3A/HDAC1 repressor complex and histone deacetylase to neuronal genes^50^. Our research further clarified that Hedgehog-Glioma-associated oncogene homolog (Hedgehog-Gli) pathway might be involved in the effect of REST on the pathogenesis of glioma. Abnormal activation of Hedgehog-Gli pathway has been proven to link to various types of cancer, including those of the brain, breast, gastrointestinal tract and blood^51–53^. Thus, the effect of REST on Hedgehog-Gli pathway during the pathogenesis of glioma requires further investigation.

In conclusion, we conducted comprehensive pan-cancer analyses of REST, revealing that REST was differentially expressed between tumor and normal tissues and acted as an independent prognostic indicator of various tumors, especially glioma. Moreover, REST expression was positively associated with immune cell infiltration and biomarkers of immune cells in glioma, as well as the immune checkpoints PD1, PD-L1, and CTLA-4, indicating its impact on tumor immunity. The molecular mechanism of REST effect on the pathogenesis of glioma was also analyzed. MiR-9-5p and miR-105-5p were the potential upstream regulatory miRNAs of REST in glioma and their upregulation were positively linked to patients’ prognosis. Importantly, the effective regulation of miR-9-5p and miR-105-5p on REST expression and the proliferation and migration of glioma cells were experimentally validated. Furthermore, HDAC1 was the potential REST-binding protein and the REST expression-correlated gene in glioma. In addition, Hedgehog-Gli pathway might be involved in the effect of REST on the pathogenesis of glioma. These findings may help to elucidate the role of REST in tumorigenesis and development of glioma and can provide a preliminary scientific basis for future clinical biomarkers and target immunotherapy studies. Our study, on the other hand, is mainly based on public databases and bioinformatics analysis and needs more experimental validation in the laboratory or in clinic. Therefore, further study is needed to validate the prognostic value of REST and investigate the underlying molecular mechanisms in tumorigenesis and development of glioma.

## Supplementary Information

Supplementary Information.

## Data Availability

The datasets generated and/or analyzed during the current study are respectively available in the free and open public databases, such as the TCGA (dbGaP Study Accession: phs000178, https://portal.gdc.cancer.gov/repository), GTEx (dbGaP Accession:phs000424.v8.p2 https://gtexportal.org/home/), GEO (GEO accession:GSE68848, https://www.ncbi.nlm.nih.gov/geo/), CPTAC (PDC study ID:PDC000204, https://proteomic.datacommons.cancer.gov/pdc) and CGGA (DataSet ID: mRNAseq_693, http://www.cgga.org.cn/) repositories. The data used to support the findings of this study are also available from the corresponding author upon request. All databases were cited and all methods were carried out in accordance with relevant guidelines and regulations.
