# Supplementary material for: Systematic analysis identifies REST as an oncogenic and immunological biomarker in glioma
Source: Sci Rep. 2023 Feb 21;13:3023. doi: 10.1038/s41598-023-30248-0 (PMC9944962; doi:10.1038/s41598-023-30248-0)

**Systematic Analysis Identifies REST as an Oncogenic and Immunological Biomarker in Glioma**

**Authors:** Guan Wang ^a^, Xiaxin Yang ^b^, Mei Qi ^c^, Meng Li ^a^, Meng Dong ^a^, Rui Xu ^a^, and Chen Zhang ^a*^

**Institutional affiliations:**

^a^ Department of Pediatrics, Qilu Hospital of Shandong University, No.107 West Wenhua Road, Jinan, 250012, Shandong Province, China

^b^ Department of Neurology, Qilu Hospital of Shandong University, No.107 West Wenhua Road, Jinan, 250012, Shandong Province, China

^c^ Department of Pathology, Qilu Hospital of Shandong University, No.107 West Wenhua Road, Jinan, 250012, Shandong Province, China

***Correspondence Author:**

Chen Zhang, Department of Pediatrics, Qilu Hospital of Shandong University, No.107 West Wenhua Road, Jinan, 250012, Shandong Province, China

Email: zczc_8888@163.com

**Supplementary material**

**Figure1** The essential information and staining intensity evaluation of immunohistochemistry (IHC) images from the Human Protein Atlas (HPA) database. **
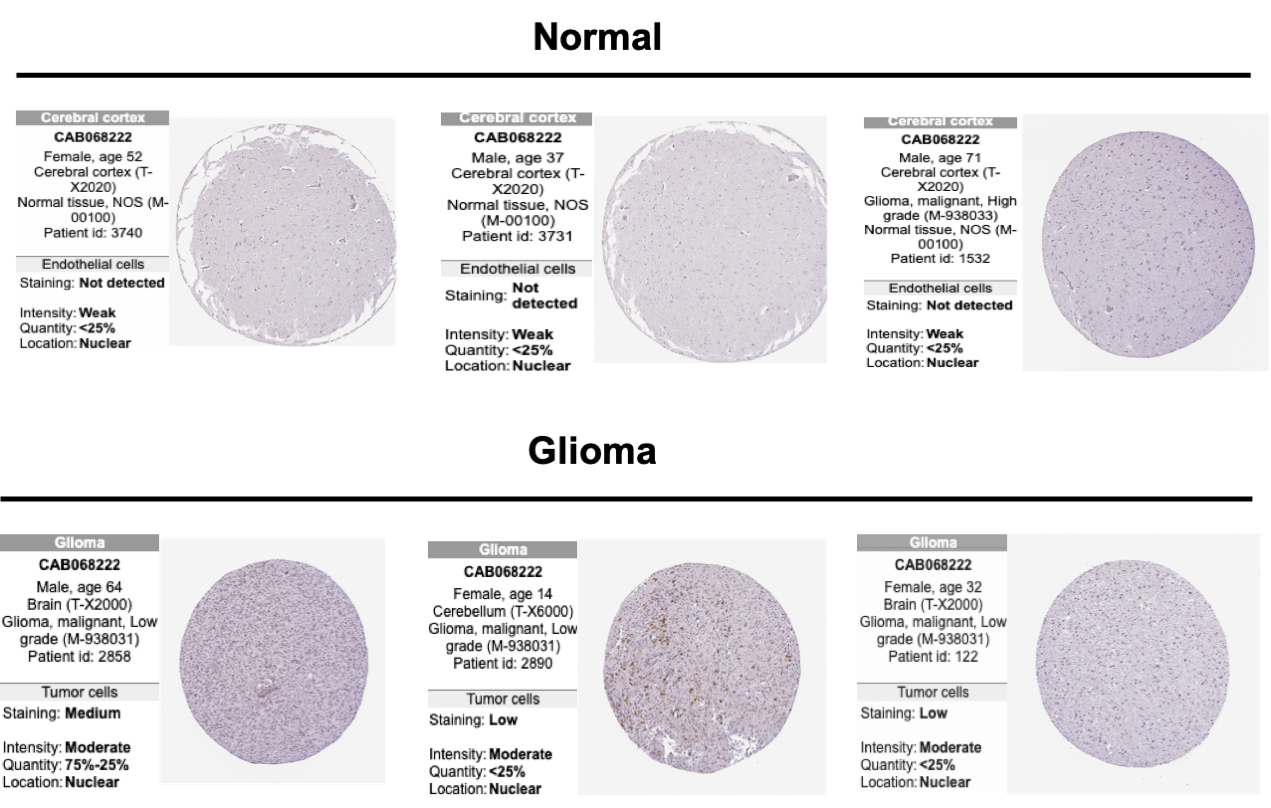
**

**Figure 2** The prognostic value of predicted miRNAs in glioma assessed by Kaplan-Meier plotter.


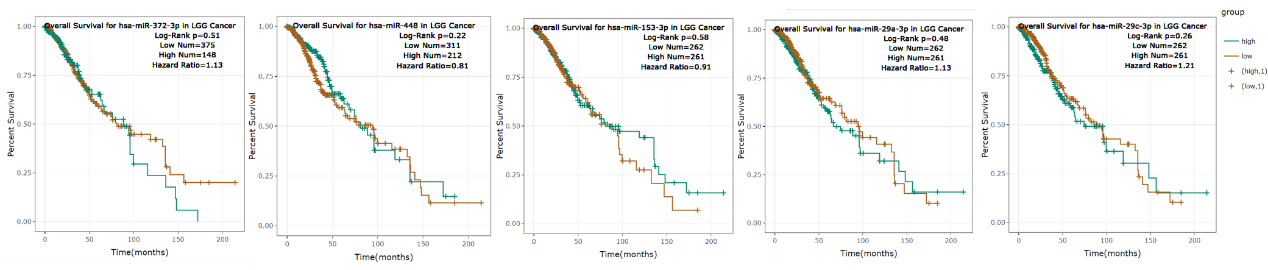


**Table 1** Tumor abbreviations.


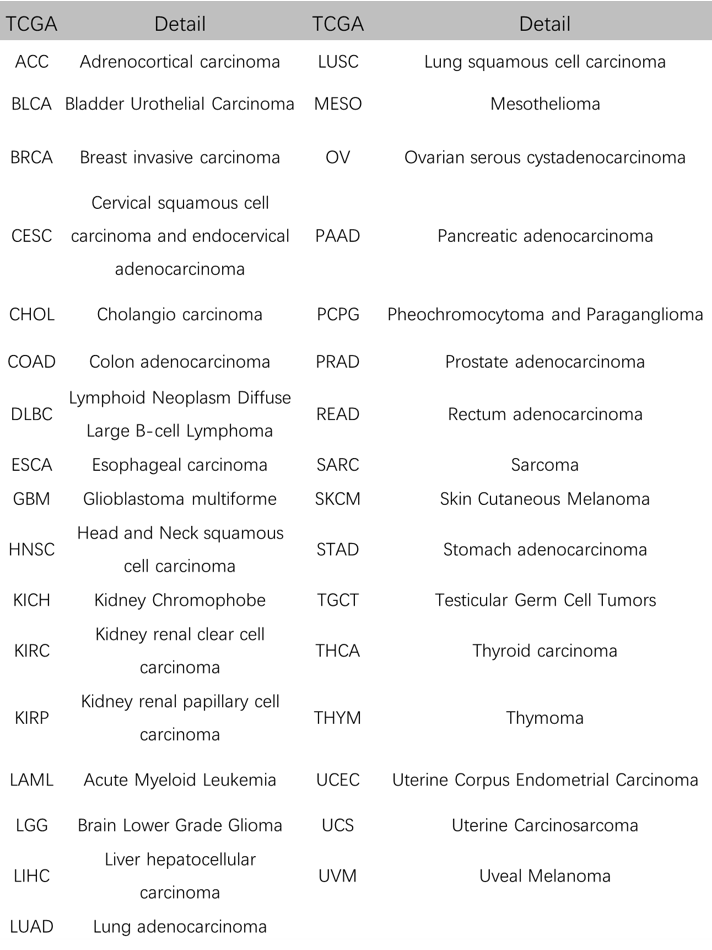


**Raw Data Images**

**Figure 7H in the main text:** Western blots were cut prior to antibody hybridization.


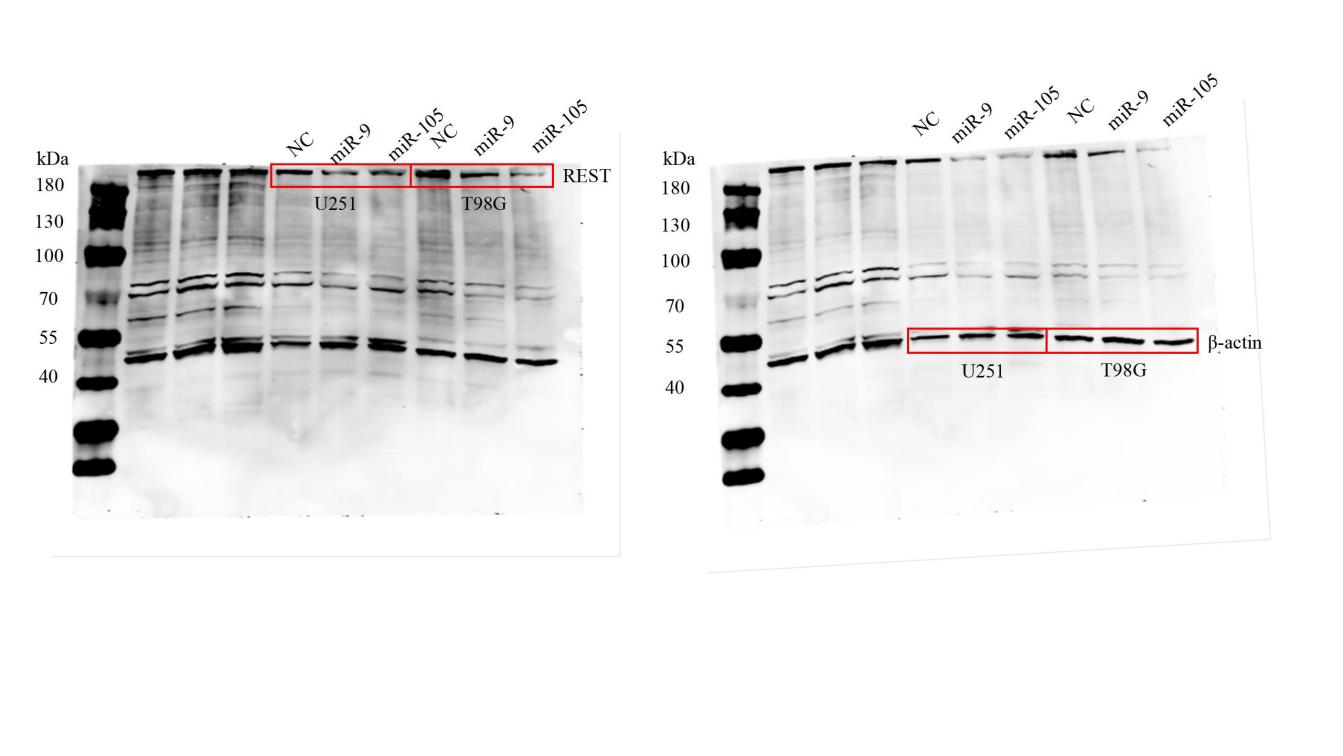

Supplement: Supplementary file 1 — Supplementary Information. [file 41598_2023_30248_MOESM1_ESM.docx]
